# Draft Genome Assemblages of 10 Xanthomonas vasicola pv. zeae Strains, Pathogens Causing Leaf Streak Disease of Maize in South Africa

**DOI:** 10.1128/genomeA.00532-18

**Published:** 2018-06-28

**Authors:** Tomasz J. Sanko, Astrid S. Kraemer, Niklaas Niemann, Arvind K. Gupta, Bradley C. Flett, Charlotte Mienie, Carlos C. Bezuidenhout

**Affiliations:** a Unit for Environmental Sciences and Management, North-West University, Potchefstroom, South Africa; b Agricultural Research Council, Potchefstroom, South Africa

## Abstract

Maize bacterial leaf streak disease has spread across maize crops in South Africa and therefore potentially poses a threat to maize production and food security. Until recently, this pathogen was identified as a Xanthomonas campestris pathovar, whereas our South African genomes seem to be more divergent and create their own subclade.

## GENOME ANNOUNCEMENT

Bacteria from the Xanthomonas genus are plant pathogens of many economically important crops. The species are divided into two main phylogenetic groups based on 16S rRNA gene and *gyrB* sequence similarity analysis (1, 2), and they are subdivided into pathovars corresponding to host plant species. The maize bacterial leaf streak disease (BLSD) pathogen was officially reported for the first time in South Africa in 1978 (3) as Xanthomonas campestris pv. vasculorum and in 1990 was renamed to X. campestris pv. zeae (4).

Our recent 16S rRNA gene results seem to suggest slow speciation of the South African BLSD maize pathogen from other Xanthomonas species. Therefore, we have addressed the question of how nucleotide composition of our whole genomes varies from those of X. campestris pathovars, with emphasis on divergence from X. campestris pv. vasculorum and X. campestris pv. zeae.

Maize leaves displaying characteristic longitudinal streak symptoms were collected from warm dry maize production regions of South Africa (mainly North West, Free State, Northern Cape, and Gauteng provinces) from nonirrigated maize fields. Tissue surfaces were sterilized and crushed in a mortar and plated on GYC agar (incubation period of 72 h at 28°C). DNA was extracted directly from pure bacterial colonies.

Initial bacterial identification was conducted using PCR with the universal ribosomal gene (16S rRNA gene) primers 27F and 1492R. Then, a 16S rRNA gene neighbor-joining (NJ) tree (default settings with maximum composite likelihood model and bootstrap calculation of 1,000 repeats) for 47 of our samples and some Xanthomonas sp. reference fragments from GenBank was drawn in MEGA7 (5).

Analysis of the NJ phylogenetic tree allowed us to select 10 representative genomes for next-generation sequencing (NGS) and 2 reference genomes, those of X. campestris and Xanthomonas axonopodis. The total DNA isolated during the preliminary trials was used for 250-bp paired-end sequencing (North-West University, Potchefstroom campus, South Africa) on a MiSeq sequencer (Illumina). The raw reads were assessed for quality-based trimming and filtering in Trimmomatic (version 0.36) (6). The remaining read pairs were assembled using SPAdes version 3.9.0 (7). Open reading frames and RNA genes were identified by Prokka (8).

The total number of contigs varied from 124 to 184, with the largest being 436,461 bp and 387,017 bp and *N*_50_ values of 114,775 bp and 111,104 bp, respectively. The draft total genome sizes varied between 4.37 Mbp and 4.98 Mbp (GC content, 63.2%). The annotation process identified 53 to 54 tRNAs, 3 types of rRNA genes (there are two loci for 16S rRNA genes in each genome) and 4,232 coding sequences (CDS) on average.

Alignment comparisons of the 16S rRNA genes for each of the 10 South African draft genomes to the simultaneously sequenced X. campestris genome, together with some reference fragments derived from GenBank, suggest that this pathogen belongs to the X. campestris group but still forms its own sisterhood subclade to X. campestris pathovars and to X. vasicola pathovars (especially to X. vasicola pv. vasculorum). Therefore, we suggest it be renamed X. vasicola pv. zeae.

### Accession number(s).

This draft genome assembly has been deposited at GenBank under the following accession numbers: Xanthomonas vasicola pv. zeae strain X01, QCXJ00000000; X. vasicola pv. zeae strain X02, QCXI00000000; X. vasicola pv. zeae strain X09, QCXH00000000; X. vasicola pv. zeae strain X15, QCXG00000000; X. vasicola pv. zeae strain X22, QCXF00000000; X. vasicola pv. zeae strain X23, QCXE00000000; X. vasicola pv. zeae strain X45, QCXD00000000; X. vasicola pv. zeae strain XGP, QCXC00000000; X. vasicola pv. zeae strain XZ2, QCXB00000000; X. vasicola pv. zeae strain XZ9, QCXA00000000; Xanthomonas campestris strain Xc86, QCWZ00000000; and Xanthomonas axonopodis strain Xa85, QCWY00000000.
